# Predictive Factors for Hypertrophy of the Future Liver Remnant After Portal Vein Embolization: A Systematic Review

**DOI:** 10.1007/s00270-021-02877-3

**Published:** 2021-06-17

**Authors:** E. A. Soykan, B. M. Aarts, M. Lopez-Yurda, K. F. D. Kuhlmann, J. I. Erdmann, N. Kok, K. P. van Lienden, E. A. Wilthagen, R. G. H. Beets-Tan, O. M. van Delden, F. M. Gomez, E. G. Klompenhouwer

**Affiliations:** 1 Department of Radiology and Nuclear Medicine, Cancer Center Amsterdam, Amsterdam UMC, University of Amsterdam, Amsterdam, The Netherlands; 2 Department of Radiology, The Netherlands Cancer Institute, Amsterdam, The Netherlands; 3 Department of Biometrics, The Netherlands Cancer Institute, Amsterdam, The Netherlands; 4 Department of Surgical Oncology, Netherlands Cancer Institute, Amsterdam, The Netherlands; 5 Department of Surgery, Cancer Center Amsterdam, Amsterdam UMC, University of Amsterdam, Amsterdam, The Netherlands; 6 Scientific Information Service, The Netherlands Cancer Institute, Amsterdam, The Netherlands; 7 GROW School for Oncology and Developmental Biology, Maastricht University Medical Centre, Maastricht, The Netherlands; 8 Department of Interventional Radiology, Hospital Clinic Universitari de Barcelona, Barcelona, Spain

**Keywords:** Portal vein embolization (PVE), Future liver remnant (FLR), Hypertrophy

## Abstract

This systematic review was conducted to determine factors that are associated with the degree of hypertrophy of the future liver remnant following portal vein embolization. An extensive search on September 15, 2020, and subsequent literature screening resulted in the inclusion of forty-eight articles with 3368 patients in qualitative analysis, of which 18 studies were included in quantitative synthesis. Meta-analyses based on a limited number of studies showed an increase in hypertrophy response when additional embolization of segment 4 was performed (pooled difference of medians = − 3.47, 95% CI − 5.51 to − 1.43) and the use of N-butyl cyanoacrylate for portal vein embolization induced more hypertrophy than polyvinyl alcohol (pooled standardized mean difference (SMD) = 0.60, 95% CI 0.30 to 0.91). There was no indication of a difference in degree of hypertrophy between patients who received neo-adjuvant chemotherapy and those who did not receive pre-procedural systemic therapy

(pooled SMD = − 0.37, 95% CI − 1.35 to 0.61), or between male and female patients (pooled SMD = 0.19, 95% CI − 0.12 to 0.50).

The study was registered in the International Prospective Register of Systematic Reviews on April 28, 2020 (CRD42020175708).

**Supplementary Information:**

The online version contains supplementary material available at 10.1007/s00270-021-02877-3.

## Introduction

Resection of liver tumors plays a central role in the treatment of primary malignancies of the liver and colorectal liver metastases. The most important prerequisite for a safe resection is the presence of an adequate future liver remnant (FLR) that is sufficient to sustain liver function. Postoperative liver failure is still the leading cause of death following major (> 3 segments) liver resection [1].

Several methods to increase the FLR volume and function are available. Hypertrophy of the contralateral liver lobe after vascular obliteration of the hepatic vessels was first identified by James Cantlie in the nineteenth century [2], but the first pre-operative portal vein embolization (PVE) was not performed until 1984 [3]. The success of this minimally invasive procedure, and other more invasive liver augmenting techniques such as Associated Liver Partition and Portal Vein Ligation for Staged Hepatectomy (ALPPS) to increase the volume and improve the hepatic functional reserve of the FLR tissue prior to resection has allowed for more extensive liver resections and has reduced the risk of postoperative morbidity and mortality [4, 5].

The minimum absolute liver volume necessary to support hepatic function after major liver resection has not been clearly defined. However, a FLR/total liver volume (TLV) ratio of at least 25–30% is recommended in patients with otherwise normal livers and a ratio up to 40% in patients with a compromised liver function [6]. When the FLR/TELV ratio is below these levels, PVE may be performed in an attempt to increase FLR volume.

Identifying factors that predict the degree of hypertrophy of the FLR (i.e., increase in FLR/TLV ratio) after PVE, can improve the selection of patients receiving PVE and more adequately stratify patients as potential surgical candidates. The aim of this systematic review was to find predictive factors for hypertrophy of the FLR after PVE.

## Methods

### Search Strategy

This review is registered in the International Prospective Register of Systematic Reviews (CRD42020175708). The databases Pubmed/Medline, Embase (ovid) and SCOPUS were searched on July 24, 2019. On September 15, 2020, an update was performed using the same search strategy. The search included the MESH terms “Liver”, “Hypertrophy” and “Embolization, Therapeutic” (Appendix 1).

### Inclusion Criteria

Included studies had to meet the following criteria: original research papers, prospective or retrospective studies. The studies had to provide data on factors affecting FLR hypertrophy.

### Exclusion Criteria

All case reports and cohort studies reporting results in ten patients or less were excluded; also, in vitro and animal studies, reports concerning surgical approaches and patients with solely arterial embolization were not included. Papers were also excluded when there was overlap with previously published data from the same study group.

### Literature Screening

Articles were electronically downloaded into reference management software (*Rayyan QCRI and EndNote X7*) and duplicated articles were electronically or manually excluded using the Bramer method [7]. Abstracts of the remaining articles were screened by two independent investigators (EAS, BMA) using predefined criteria. Full-text versions of potentially relevant articles were subsequently reviewed by the two investigators and data were extracted. Discrepancy was solved by consensus.

### Quality Assessment and Data Extraction

The study quality was assessed by two independent reviewers using the Joana Briggs Institute (JBI) Checklist for Randomized Controlled Trials and Cohort Studies. All information was independently extracted and cross-checked by two investigators according to a standard format as follows: author, publication year, country, study design, population characteristics, PVE segments and technique, FLR volumes pre- and post-PVE, factors affecting FLR hypertrophy, and completion of planned hepatic resection.

### Quantitative Synthesis

Standard fixed and random effects model estimates for the meta-analysis of continuous outcomes [8] were calculated for predictive factors for which this was possible and more than two studies could be included. The standardized mean difference (SDM) was calculated, inverse variance weighting was used for pooling and forest plots were constructed. When only median and either range or interquartile range (IQR) were available, mean and standard deviation were estimated [9]. If most studies only reported the sample median and range/IQR of the outcome, the quantile estimation method from McGrath et al. [10] was used. Heterogeneity between studies was assessed using the Q statistic (variation around the average), τ^2^ (between-study variance), H and I^2^ (percentage of variation reflecting real differences in effect size). Forest plots displayed I^2^, τ^2^ and the p value for the heterogeneity test of Q. If heterogeneity was deemed low (primarily based on a reference cut-off of 25% for I^2^ and an assessment of τ^2^), a fixed-effects model was considered appropriate. R package meta was used to perform the analyses (R version 4.0.2).

## Results

**Fig. 1:**
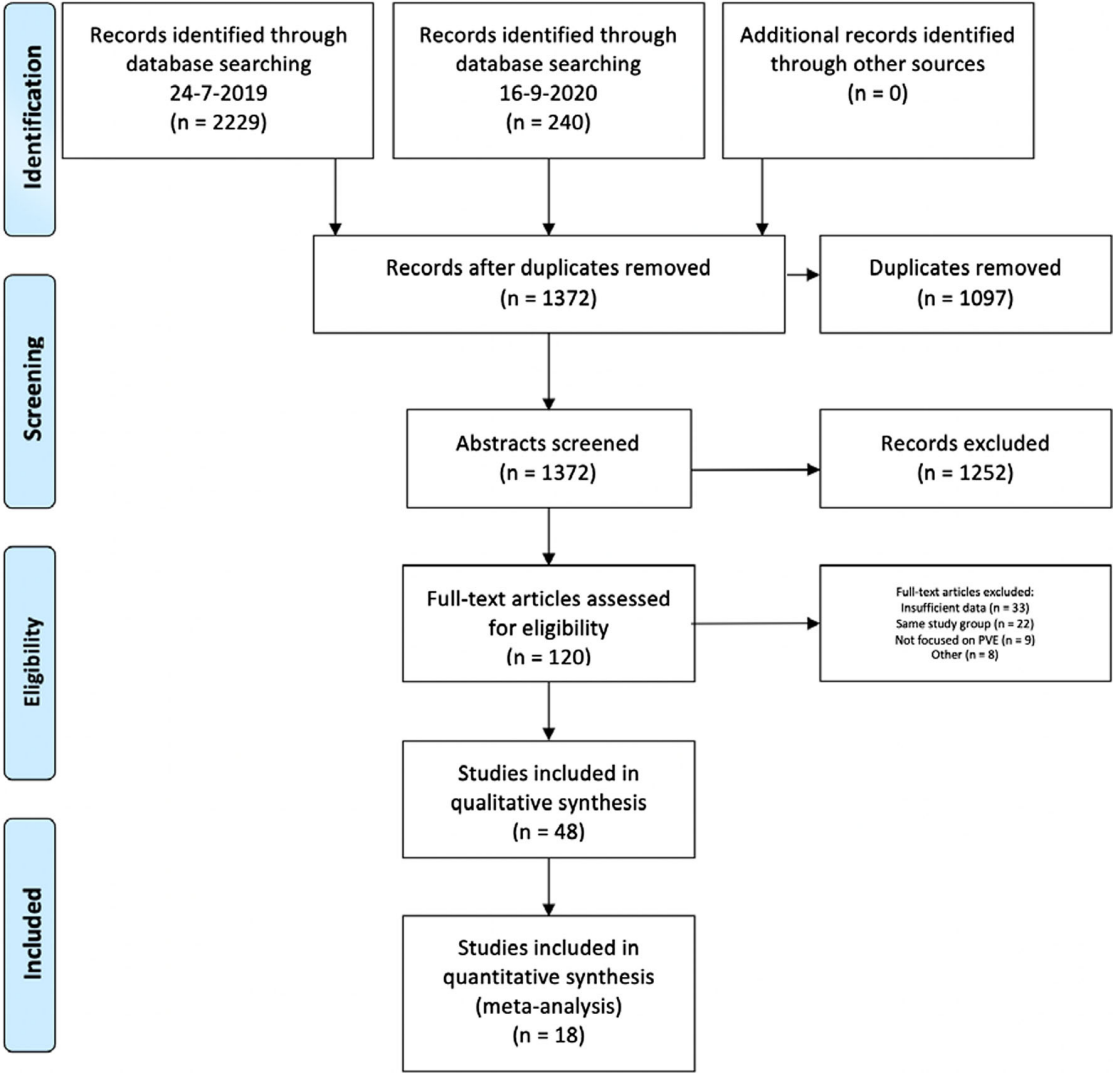
PRISMA flow diagram

The initial search resulted in 2469 records, of which 120 full-text articles were evaluated and 48 publications were included in qualitative synthesis (Fig. 1). Except for one randomized controlled trial [11], all other included studies had a retrospective study design. The quality of the literature evaluated according to the JBI grades of recommendation showed a ‘Grade A’ in six studies [11–16] and ‘Grade B’ in the remaining 42 studies [17–58].

The included papers described 3368 patients who underwent pre-operative PVE. The mean age was 62 years (range 17–87). The majority of the patients were men (65.0%). Colorectal liver metastasis (43.8%), cholangiocarcinoma (29.5%), hepatocellular carcinoma (15.2%), and gallbladder carcinoma (2.1%) were the most frequent diagnosis.

PVE approach was mainly transhepatic (81.1%) with n-butyl cyanoacrylate (NBCA (mixture of 1:1–10 with lipiodol), polyvinyl alcohol (PVA, 100–1000 μm) or a combination of these agents with coils or plugs.

2143/3368 (63.6%) patients underwent liver surgery after PVE (Appendix 2). The mean time interval between PVE and liver surgery was 43.2 days (range 23–77). Of 18.7% it was not stated whether subsequent hepatectomy was performed. 17.7% did not undergo surgery, of which 12.9% due to insufficient hypertrophy of the FLR.

## Predictive Factors for Hypertrophy of the FLR

Pre- and post-procedural computed tomography (CT) scans were performed to measure the hypertrophy response. In most studies, the absolute volumes were used to calculate the FLR with the formula: (future liver remnant volume (FLRV)/total liver volume (TLV) − tumor volume) × 100%) [12, 13, 15, 16, 18, 21–23, 28, 34–37, 40–42, 44, 45, 50, 54, 55, 58]. In other studies, TELV was calculated using CT volumetry and body surface area [14, 19, 20, 25, 29, 31, 38, 39, 53, 56, 57]. The mean time interval between PVE and post-procedural imaging for hypertrophy response was 28.5 days (range 14–56). Identified factors as potential predictors for hypertrophy response of the FLR included embolization-related factors, patient characteristics, quantitative liver function assessment, background liver disease, tumor-related factors and chemotherapy (Table 1). Eighteen studies including 1122 patients were eligible for meta-analysis [14, 16, 18, 20, 22–24, 26, 28–31, 38, 41, 48, 49], which included the factors: “*Initial FLR volume*”, “*Additional embolization of segment 4*”, “*Embolic agent**”,* “*Chemotherapy*” and “*Gender*”. For each of the remaining factors, only two or less studies reported quantitative information for carrying out a meta-analysis, and this was in consequence not performed.

**Table 1:** Predictive factors for hypertrophy of the FLR

	Study		*n* total	*n* subgroup	Univariate analysis (*p*-value)	Mutlivariate analysis (*p*-value)
*Embolization related factors*						
Initial FLR volume	Hocquelet et al.	2018	56		< 0.001*	< 0.001*
	Kasai et al.	2013	59		–	< 0.001*
	Malinowski et al.	2015	77		0.006*	0.043*
	Yamashita et al.	2017	338		–	0.034*
	De Baere et al.	2010	107		< 0.0001*	–
	Denys et al.	2005	40		< 0.05*	–
	Hammond et al.	2019	60		0.006*	–
	Igami et al.	2014	154		< 0.0001*	–
	Luz et al.	2017	50		0.017*	–
	Simoneau et al.	2016	141		< 0.001*	–
	Takahashi et al.	2019	33		0.01*	–
	Wakabayashi et al.	2002	43		0.038–0.048*	–
	Watanabe et al.	2018	152		< 0.001*	–
				*RPVE* + *S4*		
RPVE + S4	Ito et al.	2020	56	28	0.010*	0.038*
	Bjornsson et al.	2020	91	32	0.010*	–
	Hammond et al.	2019	60	22	0.011*	–
	De Baere et al.	2010	107	37	NS	
	Massimino et al.	2011	23	12	NS	
	Zeile et al.	2016	28	4	NS	
Embolic agent	Dhaliwal et al.	2018	77		0.007*	
	Guiu et al.	2013	34		< 0.05*	
	Jaberi et al.	2016	85		0.018*	
				*TACE* +		
TACE	Terasawa et al.	2020	51	23	0.035	
				*Collaterals* +		
Portal collaterals	Kohno et al.	2020	79	–	–	< 0.001*
	Zeile et al.	2016	28	7	0.004*	–
*Patient characteristics*						
Gender	De Baere et al.	2010	107		NS	
	Denys et al.	2005	40		NS	
	Dhaliwal et al.	2018	77		NS	
	Hocquelet et al.	2018	56		NS	
	Igami et al.	2014	154		NS	
	Kaido et al.	2003	46		NS	
	Malinowski et al.	2015	77		NS	
	Mise et al.	2016	332		NS	
	Nanashima et al.	2010	24		NS	
	Narita et al.	2010	42		NS	
	Rassam et al.	2019	90		NS	
	Sakakibara et al.	2014	36		NS	
	Simoneau et al.	2016	141		NS	
	Treska et al.	2013	38		NS	
	Watanabe et al.	2018	152		NS	
	Yamashita et al.	2017	338		NS	
	Yim et al.	2019	87		NS	
	Zeile et al.	2016	28		NS	
Age	Kasai et al.	2013	59		–	0.015*
	Yamashita et al.	2017	338		0.029*	0.036*
	De Baere et al.	2010	107		NS	
	Denys et al.	2005	40		NS	
	Dhaliwal et al.	2018	77		NS	
	Hocquelet et al.	2018	56		NS	
	Igami et al.	2014	154		NS	
	Kaido et al.	2003	46		NS	
	Kohno et al.	2020	79		NS	
	Malinowski et al.	2015	77		NS	
	Nanashima et al.	2010	24		NS	
	Narita et al.	2010	42		NS	
	Rassam et al.	2019	90		NS	
	Sakakibara et al.	2014	36		NS	
	Simoneau et al.	2016	141		NS	
	Takahashi et al.	2019	33		NS	
	Treska et al.	2013	38		NS	
	Wakabayashi et al.	2002	17		NS	
	Watanabe et al.	2018	152		NS	
	Yim et al.	2019	87		NS	
	Zeile et al.	2016	28		NS	
				*DM* +		
DM	Deiployi et al.	2017	76	–	NS	
	Denys et al.	2005	40	–	NS	
	Kaido et al.	2003	46	–	NS	
	Mise et al.	2016	332	50	NS	
	Narita et al.	2010	42	–	NS	
	Sakakibara et al.	2014	36	11	NS	
	Yamashita et al.	2017	338	160	NS	
	Zeile et al.	2016	28	6	NS	
				*Sarcopenia* +		
Sarcopenia	Denbo et al.	2020	45	18	0.009*	–
	Schulze et al.	2020	42	–	0.001*	–
*Quantitative liver function assessment*						
ICG	Kaido et al.	2003	46		0.010*	0.039*
	De Baere et al.	2010	107		NS	
	Igami et al.	2014	154		NS	
	Kasai et al.	2013	59		NS	
	Kohno et al.	2020	79		NS	
	Nanashima et al.	2010	24		NS	
	Sakakibara et al.	2014	34		NS	
	Treska et al.	2013	38		NS	
HBS	Rassam et al.	2019	90		NS	
*Background liver disease*						
Fibrosis	Hocquelet et al.	2018	56		0.003*	–
	Denys et al.	2005	40		0.0407*	–
				*Cirrhosis* +		
Cirrhosis	Deiployi et al.	2017	76	–	NS	
	Dhaliwal et al.	2018	77	3	NS	
	Jaberi et al.	2016	85	18	NS	
	Nanashima et al.	2006	24	2	NS	
	Sun et al.	2018	21	12	NS	
	Zeile et al.	2016	28	3	NS	
				*Hepatitis B/C* +		
Hepatitis B/C	Mise et al.	2016	332	18	NS	
	Nanashima et al.	2010	24	3	NS	
	Watanabe et al.	2018	152	19	NS	
	Yamashita et al.	2017	338	61	NS	
*Tumor related factors*						
Tumor type	De Baere et al.	2010	107		NS	
	Malinowski et al.	2015	77		NS	
	Mise et al.	2016	332		NS	
	Rassam et al.	2019	90		NS	
	Yamashita et al.	2017	319		NS	
Tumor burden	Takahashi et al.	2019	33		0.002*	–
	Treska et al.	2013	38		< 0.03*	–
				*EHD* +		
Extrahepatic CLM	Treska et al.	2013	38	17	NS	
*Chemotherapy*				*Ct* +		
	Beal et al.	2006	15	10	0.016*	–
	Kasai et al.	2013	59	7	–	0.007*
	Treska et al.	2013	38	9	< 0.03*	–
	De Baere et al.	2010	107	97	NS	
	Covey et al.	2008	100	43	NS	
	Deiployi et al.	2017	78	9	NS	
	Dhaliwal et al.	2018	77	65	NS	
	Kohno et al.	2020	79	19	NS	
	Mise et al.	2016	332	228	NS	
	Nafidi et al.	2009	20	13	NS	
	Rassam et al.	2018	90	40	NS	
	Simoneau et al.	2016	141	66	NS	
	Takahashi et al.	2019	33	14	NS	
	Tanaka et al.	2010	38	14	NS	
	Watanabe et al.	2018	152	29	NS	
	Zeile et al.	2016	28	25	NS	

FLR: future liver remnant; RPVE + S4: right portal vein embolization with segment 4; BMSC: bone marrow stem cell infusion; TACE: transarterial chemo–embolization; DM: diabetes mellitus; ICG: indocyanine green clearance test; HBS: hepatobiliairy scintigraphy; CLM: colorectal liver metastases; EHD: extrahepatic disease; Ct: neo-adjuvant chemotherapy; *: significant; NS: not significant; –: not stated

### Initial FLR Volume/Additional Embolization of Segment 4

**Fig. 2:**
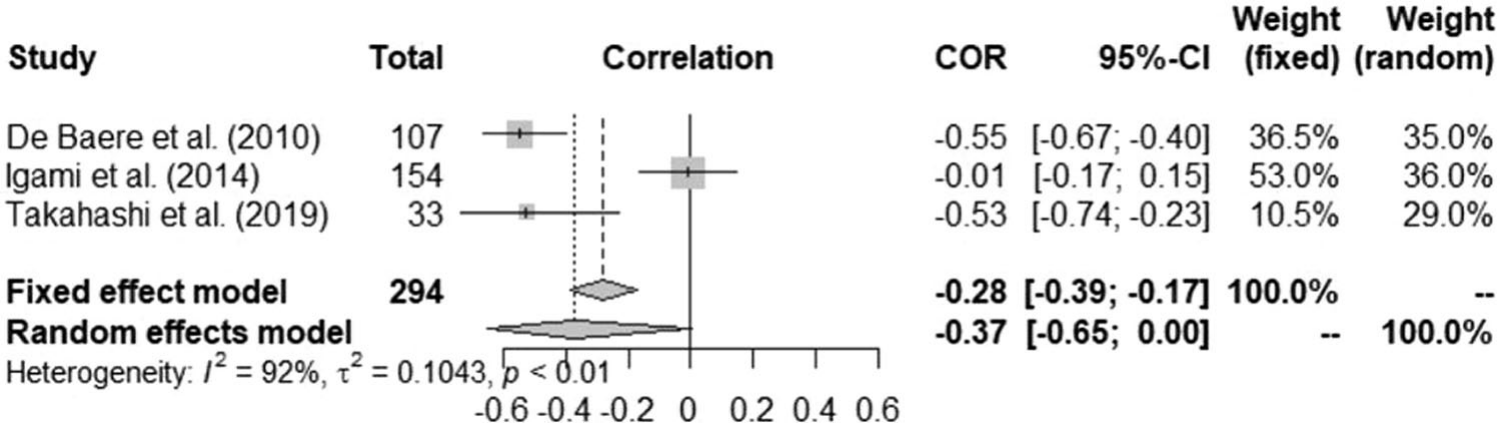
Effect of initial FLR volume on FLR hypertrophy. FLR: future liver remnant

Thirteen studies, including 1310 patients, stated that the smaller the FLR pre-PVE, the larger the FLR hypertrophy was post-PVE [12, 13, 23, 29, 30, 34, 36, 37, 46, 48, 53–55]; this inversely correlated hypertrophy response was confirmed with pooled analyses of three studies that reported correlation coefficients (pooled correlation = − 0.37, 95%CI − 0.65 to 0.00, *Fig. **2*), though with a high degree of heterogeneity (I^2^ = 92%, τ^2^ = 0.1043, p < 0.01).

**Fig. 3:**
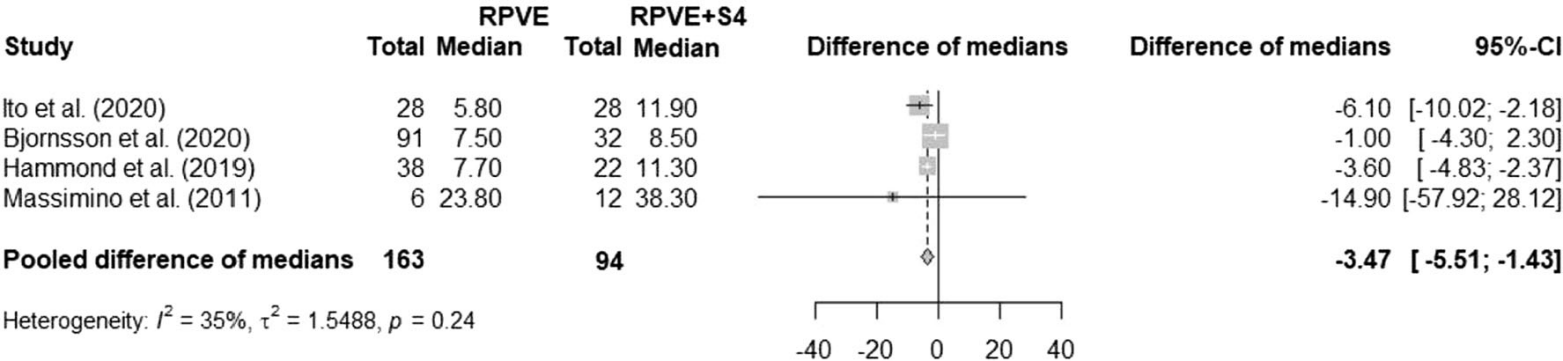
Effect of additional embolization of segment 4 on FLR hypertrophy. FLR: future liver remnant; RPVE: right portal vein embolization; S4: segment 4

When a trisectionectomy was planned, right-PVE (RPVE) with additional embolization of segment 4 (S4) was generally performed. Six studies, including a total of 365 patients, reported the effect of additional S4 embolization on the degree of hypertrophy. Three studies, with a total of 207 patients, found a significant increase in FLR hypertrophy with additional embolization of S4 [14, 20, 29]. Whereas three other studies with a total of 153 patients found no significant difference between RPVE with or without the addition of S4 [23, 38, 57]. Four studies were eligible for meta-analysis; three of these studies displayed only medians and range of degree of hypertrophy and transformations were needed to impute mean and standard deviation and obtain the standardized mean difference. It was assumed that for one study reporting only mean and standard deviation, the degree of hypertrophy distribution was assumed to be normal, and thus sample medians were estimated by the sample means and their variances were estimated by the sample variances divided by the number of subjects. When the medians and ranges were employed in the quantile estimation method [10], a difference in favor of RPVE + S4 was found (pooled difference of medians = − 3.47, 95% CI − 5.51 to − 1.43, *Fig. **3*).

### Embolic Agent

**Fig. 4:**
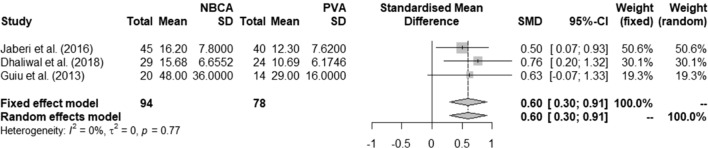
Effect of NBCA versus PVA on FLR hypertrophy. NBCA: n-butyl cyanoacrylate; PVA: polyvinyl alcohol; FLR: future liver remnant

The embolization materials mainly used were NBCA and PVA with or without coils or plugs. Three studies showed a higher hypertrophy response in 94/196 (48.0%) patients treated with NBCA ± Amplatzer-plug compared to patients treated with PVA ± coils [26, 28, 31]. The mean differences of quantitative analysis indicate that there is a significant difference of degree of hypertrophy in favor of NBCA (pooled SMD = 0.60, 95% CI 0.30 to 0.91, Fig. 4).

### Chemotherapy

Chemotherapy has potential negative side effects in the liver, most notable a non-tumoral liver parenchymal injury known as sinusoidal obstruction syndrome (SOS). There is a higher incidence of SOS in patients who received extensive (≥ 6 cycles) oxaliplatin-based chemotherapy regimens [15]. A lower hypertrophy response was seen in patients suffering from SOS (11/42, 26.2%) with an increase in the FLR of 16.8%, compared to an FLR increase of 55.6% in patients without SOS) [15]. However, in the same study, 64.3% of the patients received oxaliplatin-based neo-adjuvant chemotherapy, which showed similar hypertrophy response compared to non-oxaliplatin-based neo-adjuvant chemotherapy regimens.

**Fig. 5:**
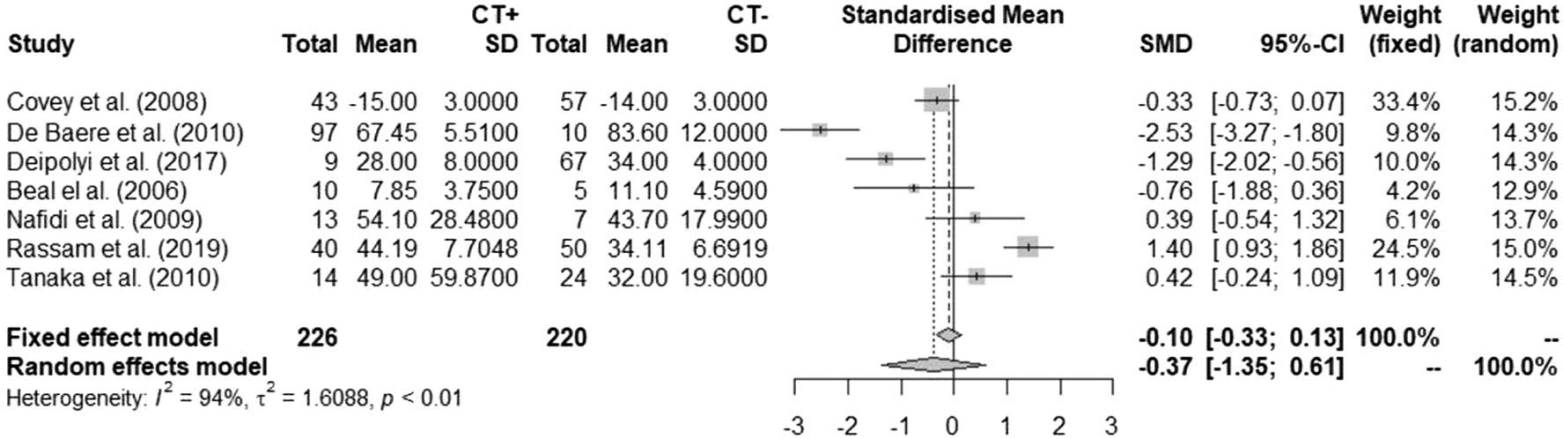
Effect of neo-adjuvant chemotherapy on FLR hypertrophy. FLR: future liver remnant; CT: chemotherapy

Regarding neo-adjuvant chemotherapy in general there are only three cohort studies that published a significant negative influence of chemotherapy on hypertrophy response [18, 34, 52]. Many other studies, including larger cohorts, could not support this finding (Table 1) [16, 22–24, 26, 27, 35, 39, 41, 46, 48, 49, 54, 57]. Pooled data showed no indication of a difference in degree of hypertrophy between patients receiving neo-adjuvant chemotherapy compared to patients who did not receive pre-procedural systemic treatment (Fig. 5). There is, however, a very high degree of heterogeneity in this relatively low number of studies (*I*^2^ = 92%, *τ*^2^ = 48.48, *p* < 0.01).

### Gender

**Fig. 6:**
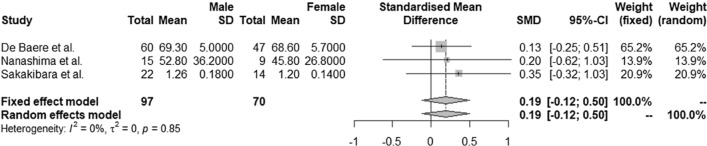
Effect of gender on FLR hypertrophy. FLR: future liver remnant

Gender was not associated with hypertrophy response in sixteen studies including 1647 patients (Table 1), which was also not significant after pooling data of three studies [23, 42, 44] with a SMD = 0.19, 95% CI − 0.12 to 0.50 (*I*^2^ = 0, *τ*^2^ = 0, *p* = 0.85, Fig. 6).

## Discussion

Although a wide range of pre-procedural factors was evaluated in the current review, only a few factors were eligible for meta-analyses, and for each of them, only a small number of studies contained the quantitative information needed for performing a meta-analysis. The included studies showed an inversely correlated hypertrophy response after PVE to the pre-embolization FLR volume: the smaller the FLR pre-PVE, the larger the FLR hypertrophy was post-PVE. Meta-analyses showed indications that the degree of hypertrophy was higher in patients with additional embolization of S4, compared to patients in whom only the right liver was embolized. Liver regeneration is a complex process, and the exact pathophysiological situation following PVE remains unclear. It is known that various cytokines, growth factors, vasoregulators, hormones and proteins initiate hepatocyte proliferation [4, 59]. It could be considered that the FLR volume will increase more if the embolized area is more extensive.

Different embolic agents have been used for PVE. NBCA and PVA, or a combination of these agents with coils/plugs is the mainly used non-absorbable materials, which lead to persistent occlusion of the portal branches, preventing peripheral recanalization. Pooled data showed a statistically significant higher degree of hypertrophy after embolization with NBCA compared to PVA ± coils. Superior increase in liver volume with NBCA plus iodized oil versus PVA plus coils was also reported in a recently published randomized controlled trial by Luz et al. [60].

Chemotherapy by means of downstaging allows patients with initially unresectable liver tumors to become resectable, which has led to an increase in exposure to chemotherapy in induction setting [61]. Previous reports showed the influence of the duration and the type of the neo-adjuvant chemotherapy on the postoperative morbidity and mortality after major hepatectomy [62, 63]; this suggests that the duration and type of chemotherapy would also affect liver regeneration after PVE. In a study by Narita et al. [15] SOS caused by oxaliplatin-based chemotherapy, inhibited FRL hypertrophy after PVE and induced postoperative liver failure. However, pooled data showed that there is no significant difference in degree of hypertrophy between patients receiving neo-adjuvant chemotherapy, including oxaliplatin-based agents, compared to patients who did not receive pre-procedural chemotherapy.

Although conventional PVE has been standard of care for the past two decades, newer approaches have been used in an attempt to increase liver hypertrophy. In patients with HCC, TACE has an anti-tumoral effect and may help to occlude arterio-portal shunts; these shunts are thought to negatively impact FLR growth [64]. Significant increase in FLR volume has been described in patients who underwent sequential TACE and PVE compared to PVE alone [50, 65], which was also noticed in a systematic review including four publications and 171 patients [66].

Novel promising liver augmenting techniques are being investigated, such as liver venous deprivation (LVD) [67]. This is a procedure in which not only the portal vein, but also the hepatic vein is embolized. The hepatic outflow obstruction induces hemodynamic changes with decrease in hepatic arterial inflow, which causes more damage to the embolized lobe [68]. This promising technique was only described in a few original research papers with limited patient numbers; therefore, these studies were not included in the current review. Guiu et al. [67] were the first to describe this technique with good results in a series of seven patients. Larger prospective trails are under way to define the role of LVD to increase the FLR.

Applications of techniques to enhance liver regeneration rely on an adequate assessment of the regenerative response of the FLR. Imaging-based volumetry is the golden standard in order to determine whether the hypertrophy response of the FLR is sufficient and safe resection can be undertaken [69]. However, volumetric assessment does not provide quantitative information of the liver. Newer imaging techniques to assess the functional share of the FLR are emerging. With HBS in combination with SPECT-CT functional and anatomic information are combined to assess segmental liver function. Using this nuclear imaging technique after PVE, showed that the functional response exceeded the volumetric response, suggesting that the waiting time to resection can be decreased [16, 70]. Functional imaging with Magnetic Resonance Imaging (MRI) with gadolinium ethoxybenzyl diethylenetriamine pentaacetic acid can also be used for the assessment regional liver function, with the advantage of characterization of liver lesions and the assessment of parenchymal disease [71, 72]. However, the assessment of liver function with MRI is still under investigation.

Radiomics uses a high throughput extraction of large amounts of quantitative imaging features with the intent of creating mineable databases from radiological images [73]. This advanced image analysis and mining of conventional medical imaging is able to capture additional information not currently used. Two previous studies showed that quantitative imaging features of the liver parenchyma correlated with hepatic insufficiency after major hepatic resection [74] and the rate of liver regeneration after liver transplantation [75]. As yet, radiomics has not been used to predict the liver hypertrophy after PVE. This innovation in medical imaging analysis might provide for biomarkers, which can be used to improve the patient selection for liver enhancing technique.

The primary limitation of this systematic review is the quality of the available literature. Most of the included articles showed a ‘Grade B’ quality according to the JBI quality assessment tool and had a retrospective design with small sample size. Due to this limited quality and the observational nature of the data, potential confounding factors could bias results. In addition, between-study heterogeneity could be influenced by the differences in inclusion criteria such as patient population, PVE technique and volumetry measurement. Besides, not all studies report which formula or method was used to measure the hypertrophy ratio. Finally, it is not clear what the criterion is in the different papers for reporting either mean and standard deviation, or median, sample size or range/IQR. For obtaining the pooled SMD with the inverse variance approach, studies reporting sample medians should either be excluded from the synthesis, or mean and standard deviation should be estimated using a transformation-based method. Applying these transformations when the data are skewed might produce biased results. This, together with the fact that only a small number of studies could be used for each factor, means that results from meta-analyses should be taken with caution.

## Conclusion

The degree of hypertrophy was found to be more pronounced when NBCA was used as embolic agent and when a larger volume was embolized. Neo-adjuvant chemotherapy and gender do not influence the degree of hypertrophy response. Due to the quality level and heterogeneity of the included studies and lack of randomized controlled trials, no other conclusions could be drawn. Techniques that may improve patient selection for a liver regenerating procedure and more adequately stratify patients as surgical candidates remain a subject of further research.

## Supplementary Information

Below is the link to the electronic supplementary material.

Supplementary file 1 (DOCX 14 kb)

Supplementary file 2 (DOCX 32 kb)
