# Supplementary material for: Predictive Factors for Hypertrophy of the Future Liver Remnant After Portal Vein Embolization: A Systematic Review
Source: Cardiovasc Intervent Radiol. 2021 Jun 17;44(9):1355–66. doi: 10.1007/s00270-021-02877-3 (PMC8382618; doi:10.1007/s00270-021-02877-3)
Supplement: Supplementary file 1 — (DOCX 14 kb) [file 270_2021_2877_MOESM1_ESM.docx]

*Appendix 1*

**Search strategy**

**PubMed (Medline)**

| N | Searches |
| --- | --- |
| #3 | #1 AND #2 |
| #2 | "Embolization, Therapeutic"[Mesh] OR “portal vein embolization”[tiab] OR “portal venous embolization”[tiab] OR PVE[tiab] OR “portal vein occlusion”[tiab] OR PVO[tiab] OR “portal vein obstruction”[tiab] |
| #1 | (("Hypertrophy"[Mesh:NoExp] OR “volume increase*”[tiab] OR “increase of volume”[tiab] OR “volume significantly increase*”[tiab] OR “growth rate*”[tiab] OR “regeneration index”[tiab] OR hypertroph*[tiab]) AND ("Liver"[Mesh] OR liver*[tiab] OR hepatic*[tiab])) OR “future liver remnant volume”[tiab] OR “FLR volume”[tiab] OR “future remnant liver growth”[tiab] OR “FRL growth”[tiab] OR "Hepatomegaly"[Mesh] OR hepatomegal*[tiab] |

**Embase (ovid)**

| N | Searches |
| --- | --- |
| #3 | 1 AND 2 |
| #2 | exp 'liver hypertrophy'/ or ("future liver remnant volume" or "FLR volume" or "future remnant liver growth" or "FRL growth").ti,ab,kw. or (("volume increase*" or "increase of volume" or "volume significantly increase*" or "growth rate*" or "regeneration index" or "hypertroph*").ti,ab,kw. and (exp 'liver'/ or (liver* or hepatic*).ti,ab,kw.)) |
| #1 | exp 'artificial embolization'/ or ("portal vein embolization" or "portal venous embolization" or "PVE" or "portal vein occlusion" or "PVO" or "portal vein obstruction").ti,ab,kw. |

**SCOPUS**

| N | Searches |
| --- | --- |
| #3 | #1 AND #2 |
| #2 | TITLE-ABS-KEY ( "future liver remnant volume" OR "FLR volume" OR "future remnant liver growth" OR "FRL growth" ) OR ( TITLE-ABS-KEY ( "volume increase*" OR "increase of volume" OR "volume significantly increase*" OR "growth rate*" OR "regeneration index" OR "hypertroph*" ) AND TITLE-ABS-KEY ( liver* OR hepatic* ) ) |
| #1 | TITLE-ABS-KEY ( "portal vein embolization" OR "portal venous embolization" OR "PVE" OR "portal vein occlusion" OR "PVO" OR "portal vein obstruction" ) |
