# Supplementary material for: Predictive Factors for Hypertrophy of the Future Liver Remnant After Portal Vein Embolization: A Systematic Review
Source: Cardiovasc Intervent Radiol. 2021 Jun 17;44(9):1355–66. doi: 10.1007/s00270-021-02877-3 (PMC8382618; doi:10.1007/s00270-021-02877-3)
Supplement: Supplementary file 2 — (DOCX 32 kb) [file 270_2021_2877_MOESM2_ESM.docx]

| *Appendix 2* | | | | | | | |  |  |  |  |  |  |  |
| --- | --- | --- | --- | --- | --- | --- | --- | --- | --- | --- | --- | --- | --- | --- |
|  | | | | | | | |  |  |  |  |  |  |  |
| **Included articles** | | | | | | | |  |  |  |  |  |  |  |
|  | | | | | |  | |  |  |  |  |  |  |  |
|  | **Study** |  | **Retrospective/**  **Prospective** | **JBI grade** | ***n* total** | ***n* catagorized** | | **Pre–PVE FLR (%)** | **Post–PVE FLR (%)** | **Pre–PVE FLR (mL)** | **Post–PVE FLR (mL)** | **Increase FLR (%)** | **DH** | **Surgical resection achieved after PVE** |
| 1 | Am Esch et al. | 2012 | Retrospective | B | 22 | 11 | BMSC– | 19.3 | 25.6 | 289.3 | 379.1 | – | 4.5 | 8/11 |
|  |  |  |  |  |  | 11 | BMSC+ | 16.9 | 29.2 | 241.4 | 411.2 | – | 10.2 | 9/11 |
| 2 | Beal et al. | 2006 | Retrospective | B | 15 |  |  | 18 | 27 | 270 | 400 | – | 9.0 | 8/15 |
| 3 | Beppu et al. | 2015 | Prospective | A | 28 | 13 | BCCAA+ | – | – | – | – | – | – | 9/13 |
|  |  |  |  |  |  | 15 | BCCAA– | – | – | – | – | – | – | 12/15 |
| 4 | Biggeman et al. | 2019 | Retrospective | B | 29 | 8 | PVA | 15.3 | 25.5 | 244.8 | 403.2 | – | – | 7/8 |
|  |  |  |  |  |  | 10 | Lipiodol+PVA | 17.9 | 25.1 | 278.6 | 391.0 | – | – | 10/10 |
|  |  |  |  |  |  | 11 | EVOH | 17.6 | 26.8 | 305.3 | 468.0 | – | – | 11/11 |
| 5 | Bjornsson et al. | 2020 | Retrospective | B | 232 | 196 | RPVE | 23.3 | 32.4 | 380.0 | 531.0 | 37.9 | 8.5 | 142/196 |
|  |  |  |  |  |  | 36 | RPVE+S4 | 19.5 | 27.3 | 333.0 | 479.0 | 47.0 | 8.0 | 21/36 |
| 6 | Capussotti et al. | 2005 | Retrospective | B | 26 | 13 | RPVE | 22 | 34 | – | – | 51.4 | – | 10/13 |
|  |  |  |  |  |  | 13 | RPVE+S4 | 19 | 29 | – | – | 54.8 | – | 10/13 |
| 7 | Covey et al. | 2008 | Retrospective | B | 100 | 57 | Ct– | – | – | – | – | 26 | – | 71/100 |
|  |  |  |  |  |  | 43 | Ct+ | – | – | – | – | 22 | – | – |
| 8 | De Baere et al. | 2010 | Retrospective | B | 107 |  |  | 24 | 37 | 344 | 543 | 69 | – | 94/107 |
| 9 | Deipolyi et al. | 2017 | Retrospective | B | 76 | 67 | Ct– | – | – | – | – | 34 | – | 53/67 |
|  |  |  |  |  |  | 9 | Ct+ | – | – | – | – | 28 | – | 8/9 |
| 10 | Denys et al. | 2005 | Retrospective | A | 40 |  |  | – | – | 492 | 655 | 41 | – | – |
| 11 | Denbo et al. | 2020 | Retrospective | B | 45 | 18 | Sarcopenic+ | 26.5 | – | – | – | – | 8.3 | – |
|  |  |  |  |  |  | 27 | Sarcopenic– | 28,8 | – | – | – | – | 15.2 | – |
| 12 | Dhaliwal et al. | 2018 | Retrospective | B | 77 | 29 | NBCA | – | – | 647.1 | 920.4 | – | 14.8 | 52/77 |
|  |  |  |  |  |  | 24 | NBCA+PVA | – | – | 514.4 | 686.7 | – | 10.1 | – |
|  |  |  |  |  |  | 24 | PVA | – | – | 501.1 | 697.4 | – | 9.3 | – |
| 13 | Farges et al. | 2003 | Prospective | B | 27 | 13 | CLD- | 31 | 47 | 442 | 626 | 44 | 16 | 13/13 |
|  |  |  |  |  |  | 14 | CLD+ | 35 | 44 | 448 | 605 | 35 | 9 | 14/14 |
| 14 | Goere et al. | 2006 | Retrospective | B | 20 | 10 | Ct– | 23.3 | 23.0 | – | – | – | – | 7/10 |
|  |  |  |  |  |  | 10 | Ct+ | 25.1 | 33.0 | – | – | – | – | 7/10 |
| 15 | Guiu et al. | 2013 | Retrospective | B | 34 | 20 | NBCA | – | – | 470 | 682 | 36 | – | – |
|  |  |  |  |  |  | 14 | SM | – | – | 495 | 600 | 29 | – | – |
| 16 | Hammond et al. | 2019 | Retrospective | B | 60 | 38 | RPVE | 17.5 | 25.2 | 280.9 | – | – | – | 32/38 |
|  |  |  |  |  |  | 22 | RPVE+S4 | 19.1 | 30.4 | 310.3 | – | – | – | 15/22 |
| 17 | Hocquelet et al. | 2018 | Retrospective | A | 56 |  |  | 31.5 | 41 | – | – | 25.6 | – | 43/56 |
| 18 | Igami et al. | 2014 | Retrospective | B | 154 |  |  | 34.4 | 44.9 | 384 | 497 | – | – | – |
| 19 | Ito et al. | 2020 | Retrospective | A | 56 | 28 | RPVE | 23.5 | 29.6 | 157 | 349 | 32.3 | 5.8 | 28/28 |
|  |  |  |  |  |  | 28 | RPVE+S4 | 26.5 | 39.8 | 173 | 426 | 52.4 | 11.9 | 28/28 |
| 20 | Jaberi et al. | 2016 | Retrospective | B | 85 | 45 | NBCA+AVP | 33 | 49.3 | 511 | 765.4 | – | – | 30/45 |
|  |  |  |  |  |  | 40 | PVA | 29.9 | 42.2 | 468 | 663.5 | – | – | 30/40 |
| 21 | Kaido et al. | 2003 | Retrospective | B | 46 |  |  | – | – | – | – | – | – | – |
| 22 | Kaneko et al. | 2002 | Retrospective | B | 23 |  |  | – | – | 411 | 513 | 25.2 | – | 18/23 |
| 23 | Kasai et al. | 2013 | Retrospective | B | 59 |  |  | 29.2 | 37.5 | 346 | 438 | 28.8 | – | 52/59 |
| 24 | Kohno et al. | 2020 | Retrospective | B | 79 |  |  | 30.3 | 38.2 | 351 | 450 | 30.9 | – | 70/79 |
| 25 | Luz et al. | 2017 | Retrospective | B | 50 |  |  | 29.6 | 42.3 | 421 | 629 | 51.7 | – | 31/50 |
| 26 | Malinowski et al. | 2015 | Retrospective | B | 77 | 20 | FLR <15% | 23 | 25 | 448.2 | 475.5 | 9 | – | 61/77 |
|  |  |  |  |  |  | 34 | FLR 15–55% | 19 | 25 | 315.2 | 400.4 | 28 | – | – |
|  |  |  |  |  |  | 23 | FLR 55% | 16 | 27 | 283.4 | 469.1 | 65 | – | – |
| 27 | Massimino et al. | 2011 | Retrospective | B | 23 | 10 | RPVE | 26.5 | 37.5 | 532.1 | 739.4 | 23.8 | – | 8/10 |
|  |  |  |  |  |  | 13 | RPVE+S4 | 23.8 | 35.7 | 441.0 | 579.6 | 38.3 | – | 12/13 |
| 28 | Mise et al. | 2016 | Retrospective | B | 332 |  |  | 14.9 | 22.4 | 256.7 | 393.3 | – | – | – |
| 29 | Miura et al. | 2019 | Retrospective | B | 76 | 29 | BD1 | 36.0 | 43.4 | 460 | 555 | – | – | 75/76 |
|  |  |  |  |  |  | 47 | BD2 | 34.9 | 43.4 | 455 | 577 | – | – | – |
| 30 | Nafidi et al. | 2009 | Retrospective | B | 20 | 7 | Ct– | 31.1 | 43.9 | – | – | 43.7 | – | 2/7 |
|  |  |  |  |  |  | 13 | Ct+ | 29.5 | 43.0 | – | – | 54.1 | – | 11/13 |
| 31 | Nanashima et al. | 2010 | Retrospective | B | 24 |  |  | 33 | 43 | – | 500 | – | – | 18/24 |
| 32 | Narita et al. | 2011 | Retrospective | A | 42 | 31 | SOS– | – | – | – | – | 55.6 | – | 32/42 |
|  |  |  |  |  |  | 11 | SOS+ | – | – | – | – | 16.8 | 7.9 |  |
| 33 | Peng et al. | 2012 | Retrospective | B | 54 | 25 | PVE alone | – | – | – | – | – | 7.4 | 19/25 |
|  |  |  |  |  |  | 29 | PVE+IAT | – | – | – | – | – | – | 27/29 |
| 34 | Rassam et al. | 2019 | Retrospective | A | 90 |  | S2-3 | 16.7 | 23.8 | 274.2 | – | 41.8 | – | 65/90 |
|  |  |  |  |  |  |  | S2-4 | 30.1 | 40.8 | 488.7 | 639.9 | 36.8 | – | – |
| 35 | Sakakibara et al. | 2014 | Retrospective | B | 36 |  |  | – | – | – | – | – | – | 30/36 |
| 36 | Schulze et al. | 2020 | Retrospective | B | 42 |  |  | 34.3 | 43.2 | 570 | 759 | – | 8.9 | 38/42 |
| 37 | Simoneau et al. | 2016 | Retrospective | B | 141 |  |  | 21.6 | 29.5 | 315 | 512 | 32.5 | – | 80/141 |
| 38 | Sun et al. | 2018 | Retrospective | B | 21 | 9 | Cirrhosis– | – | – | 447.9 | 627.2 | 45.6 | – | 21/21 |
|  |  |  |  |  |  | 12 | Cirrhosis+ | – | – | 412.4 | 549.2 | 31.1 | – | – |
| 39 | Takahashi et al. | 2019 | Retrospective | B | 33 |  |  | – | – | – | – | 33.7 | – | – |
| 40 | Tanaka et al. | 2010 | Retrospective | B | 38 | 24 | Ct– | – | – | 275 | 366 | 33 | – | 24/24 |
|  |  |  |  |  |  | 14 | Ct+ | – | – | 285 | 402 | 49 | – | 14/14 |
| 41 | Terasawa et al. | 2020 | Retrospective | B | 51 | 28 | PVE alone | – | – | 568 | 700 | 31 | – | 21/28 |
|  |  |  |  |  |  | 23 | PVE+TACE | – | – | 529 | 838 | 43 | – | 19/23 |
| 42 | Treska et al. | 2013 | Retrospective | B | 38 |  |  | – | – | – | – | – | – | 13/38 |
| 43 | Treska et al. | 2018 | Retrospective | B | 55 | 27 | PVE alone | – | – | – | – | – | – | 24/27 |
|  |  |  |  |  |  | 28 | PVE+BMSC | – | – | – | – | – | – | 23/28 |
| 44 | Wakabayashi et al. | 2002 | Retrospective | B | 43 | 17 | NLP | 27 | 36 | 295 | 395 | – | – | 17/17 |
|  |  |  |  |  |  | 26 | DLP | 32–35 | 38–41 | 369–371 | 409–487 | – | – | 26/26 |
| 45 | Watanabe et al. | 2018 | Retrospective | B | 152 |  |  | 33 | 42 | 364 | 451 | 24 | – | 152/152 |
| 46 | Yamashita et al. | 2017 | Retrospective | B | 319 | 70 | HCC | 41 | 51 | – | – | – | – | 64/70 |
|  |  |  |  |  |  | 172 | CC | 36 | 46 | – | – | – | – | 133/172 |
|  |  |  |  |  |  | 77 | CLM | 35 | 45 | – | – | – | – | 58/77 |
| 47 | Yim et al. | 2019 | Retrospective | B | 87 |  |  | 37 | 45 | 529.1 | 640.5 | – | – | 75/87 |
| 48 | Zeile et al. | 2016 | Retrospective | B | 28 |  |  | 20.3 | 32.0 | – | – | – | – | 28/28 |

JBI: Joanna Briggs Institute, PVE: portal vein embolization; FLR: future liver remnant; DH: degree of hypertrophy; BMSC: bone marrow stem cell infusion; BCCAA: branched-chain amino acid supplementation; PVA: polyvinyl alcohol; EVOH: ethylene vinyl alcohol copolymer; RPVE: right portal vein embolization; S4: segment 4; Ct: chemotherapy; NBCA: n-butyl cyanoacrylate; CLD: chronic liver disease; SM: spherical microparticles; AVP: Amplatzer vascular plug; BD1: unilateral biliairy drainage; BD2: bilateral biliairy drainage; SOS: sinusoidal obstruction syndrome; IAT: intra-arterial therapy; S2-3: segment 2-3; S2-4: segment 2-4; TACE: transarterial chemo-embolization; NLP: normal liver parenchyma; DLP: diseased liver parenchyma; HCC: hepatocellular carcinoma; CC: cholangiocarcinoma; CLM: colorectal liver metastases; –: not stated.
